# Microsurgical excision of a compressive thoracic arachnoid cyst: Technical pearls

**DOI:** 10.1002/ccr3.2420

**Published:** 2019-09-12

**Authors:** Gregory Glauser, Dmitriy Petrov, Omar Choudhri

**Affiliations:** ^1^ Department of Neurosurgery University of Pennsylvania Philadelphia Pennsylvania

**Keywords:** back pain, hydromyelia, spine, syrinx, thoracic arachnoid cyst

## Abstract

The key clinical message of this case is that it is critical to differentiate between arachnoid cysts and spinal cord herniation. This is performed by evaluating the ventral dura to assure that it remains intact.

## Graphical abstract

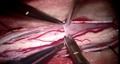

The key clinical message of this case is that it is critical to differentiate between arachnoid cysts (Figure 1) and spinal cord herniation. This is performed by evaluating the ventral dura to assure that it remains intact.^1^, ^2^

**Figure 1:**
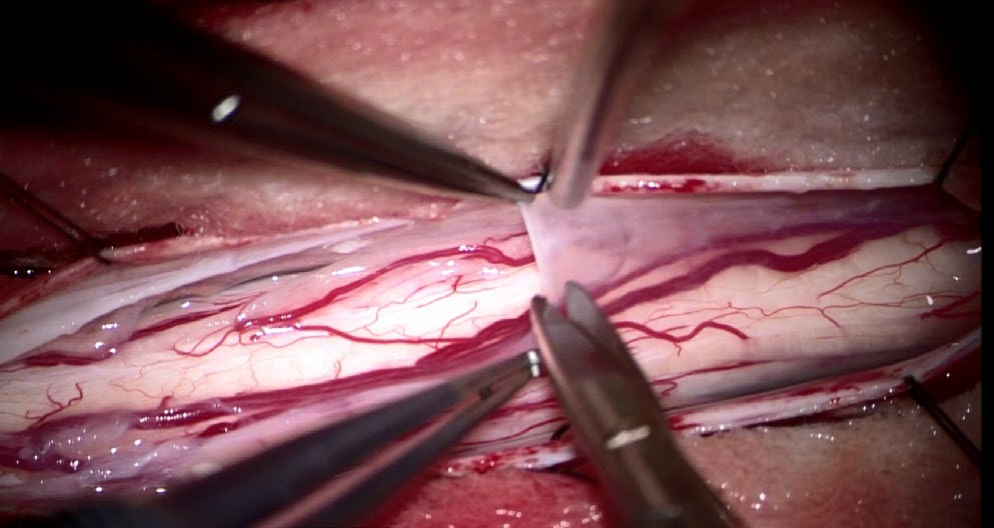
Operative view of the spinal cord during cyst excision

## AUTHOR CONTRIBUTIONS

All authors involved in preparation of the video concur that no work resembling the enclosed video has been published or is being submitted for publication elsewhere. We certify that we have each made a substantial contribution as to qualify for authorship as follows: OC and DP: performed the procedure. OC: provided video narration. GG: performed critical video editing and preparation for publication. Strict adherence to all university, state, and federal requirements regarding patient confidentiality and care has been upheld. The authors have no personal, financial, or institutional interest in any of the materials or devices described in the video.
